# Corticosterone induces discrete epigenetic signatures in the dorsal and ventral hippocampus that depend upon sex and genotype: focus on methylated *Nr3c1* gene

**DOI:** 10.1038/s41398-022-01864-7

**Published:** 2022-03-16

**Authors:** Salvatore G. Caradonna, Nathan R. Einhorn, Vikram Saudagar, Huzefa Khalil, Gordon H. Petty, Axel Lihagen, Claire LeFloch, Francis S. Lee, Huda Akil, Alessandro Guidotti, Bruce S. McEwen, Eleonora Gatta, Jordan Marrocco

**Affiliations:** 1 Laboratory of Neuroendocrinology, The Rockefeller University, New York, NY USA; 2 Department of Psychiatry, Center for Alcohol Research in Epigenetics, Psychiatric Institute, University of Illinois at Chicago, Chicago, IL USA; 3 Michigan Neuroscience Institute, University of Michigan, Ann Arbor, MI USA; 4 Department of Neuroscience, Columbia University, New York, NY USA; 5 The Health Center Vadstena, Vadstena, Sweden; 6 Department of Psychiatry, Sackler Institute for Developmental Psychobiology, Weill Cornell Medical College, New York, NY USA

**Keywords:** Epigenetics and behaviour, Molecular neuroscience

## Abstract

The genomic effects of circulating glucocorticoids are particularly relevant in cortico-limbic structures, which express a high concentration of steroid hormone receptors. To date, no studies have investigated genomic differences in hippocampal subregions, namely the dorsal (dHPC) and ventral (vHPC) hippocampus, in preclinical models treated with exogenous glucocorticoids. Chronic oral corticosterone (CORT) in mouse is a pharmacological approach that disrupts the activity of the hypothalamic-pituitary-adrenal axis, increases affective behavior, and induces genomic changes after stress in the HPC of wildtype (WT) mice and mice heterozygous for the gene coding for brain-derived neurotrophic factor Val66Met (hMet), a variant associated with genetic susceptibility to stress. Using RNA-sequencing, we investigated the genomic signatures of oral CORT in the dHPC and vHPC of WT and hMet male and female mice, and examined sex and genotype differences in response to oral CORT. Males under CORT showed lower glycemia and increased anxiety- and depression-like behavior compared to females that showed instead opposite affective behavior in response to CORT. Rank–rank-hypergeometric overlap (RRHO) was used to identify genes from a continuous gradient of significancy that were concordant across groups. RRHO showed that CORT-induced differentially expressed genes (DEGs) in WT mice and hMet mice converged in the dHPC of males and females, while in the vHPC, DEGs converged in males and diverged in females. The vHPC showed a higher number of DEGs compared to the dHPC and exhibited sex differences related to glucocorticoid receptor (GR)-binding genes and epigenetic modifiers. Methyl-DNA-immunoprecipitation in the vHPC revealed differential methylation of the exons 1_C_ and 1_F_ of the GR gene (*Nr3c1*) in hMet females. Together, we report behavioral and endocrinological sex differences in response to CORT, as well as epigenetic signatures that i) differ in the dHPC and vHPC,ii) are distinct in males and females, and iii) implicate differential methylation of *Nr3c1* selectively in hMet females.

## Introduction

Glucocorticoids exert their effects by binding to glucocorticoid receptors (GRs), which regulate up to ~20% of the genome via both direct (by binding to glucocorticoid responsive elements in promoter regions) and indirect mechanisms (by interacting with bound transcription factors and epigenetic modifiers) [1]. GRs play a key role in the feedback regulation of the hypothalamic-pituitary-adrenal (HPA) axis [2]. They are highly expressed in the hippocampus [3, 4] and their distribution is heterogeneous depending on the hippocampal subregion both at baseline [5] and in response to stress [6, 7]. The dorsal (dHPC) and the ventral hippocampus (vHPC) are two functionally distinct subregions that differ in their respective neuroanatomical connectivity and in the biological processes they encode [8, 9]. Regional differences in gene expression also contribute to distinguish the function of the dHPC and vHPC in response to environmental stimuli [10–13]. These two hippocampal circuits also show sex differences in neuronal proliferation [14, 15], indicating that males and females use distinct networks to modulate the function of the HPA axis. However, little is known on the whole-genome profile that underlies sex differences in the dHPC and vHPC, especially in response to glucocorticoids.

Chronic administration of oral corticosterone (CORT) in mice is a validated pharmacological model for the study of the molecular and behavioral consequences of HPA axis dysregulation [16, 17]. Oral CORT in mice induces hyperphagia, increased locomotion [16], and stress-induced grooming, rearing, and exploratory behavior in males [18, 19]. In females, oral CORT increases stress-induced immobility and decreases social interaction and hyponeophagia [20]. Others have shown that CORT induces decreased prepulse inhibition only in mice heterozygous (hMet) for the gene coding brain-derived neurotrophic factor (BDNF) Val66Met [21], a genetic variant that increases risk for stress-inducible pathologies [22, 23]. Notably, hMet mice show disruption of the HPA axis activity without any applied stressors [24] in association with genomic signatures in the CA3 pyramidal neurons of the hippocampus that differ from wild-type (WT) mice before and after acute stress. Sex differences are also observed in hMet mice, particularly regarding glucocorticoid-dependent networks [25, 26].

Using RNA-sequencing, RT-qPCR, and Methylated DNA Immunopreciptation (MeDIP) we investigated the epigenetic signatures in the dHPC and vHPC induced by chronic low doses of oral CORT as a function of sex and genotype. We report behavioral sex differences in response to CORT, as well as sex- and genotype-specific genomic effects in the dHPC and vHPC that included one cluster of glucocorticoid receptor-binding genes and one cluster of epigenetic modifiers and showed that CORT is associated with differential methylation of the GR gene (*Nr3c1*).

## Methods

### Animals

Mice heterozygous for the BDNF allele (hMet) were generated in the Francis Lee laboratory, as previously described [22]. C57/BL6 mice (WT) and BDNF hMet mice were obtained by performing in-house breeding. To control for litter-specific effects, mice were selected from multiple litters. Animals were group housed (*n* = 4–5) in standard cages (28.5 × 17 × 13 cm), which were changed weekly, and were kept on a 12-h light–dark cycle (lights off 7:00 pm) in a temperature-controlled room maintained at 21 ± 2 °C. A 2lux red light permitted animal maintenance in the dark phase. Food and water were available ad libitum. Based on previous behavioral studies, we found that a sample size of 8–12 mice per group allows us to reliably detect changes of the magnitude we are examining (*α* = 0.05). Variance was similar among the groups that are being compared. All procedures were performed in accordance with the National Guidelines on the Care and Use of Animals and a protocol approved by The Rockefeller University Animal Care and Use Committee.

### Chronic oral CORT treatment

**Fig. 1:**
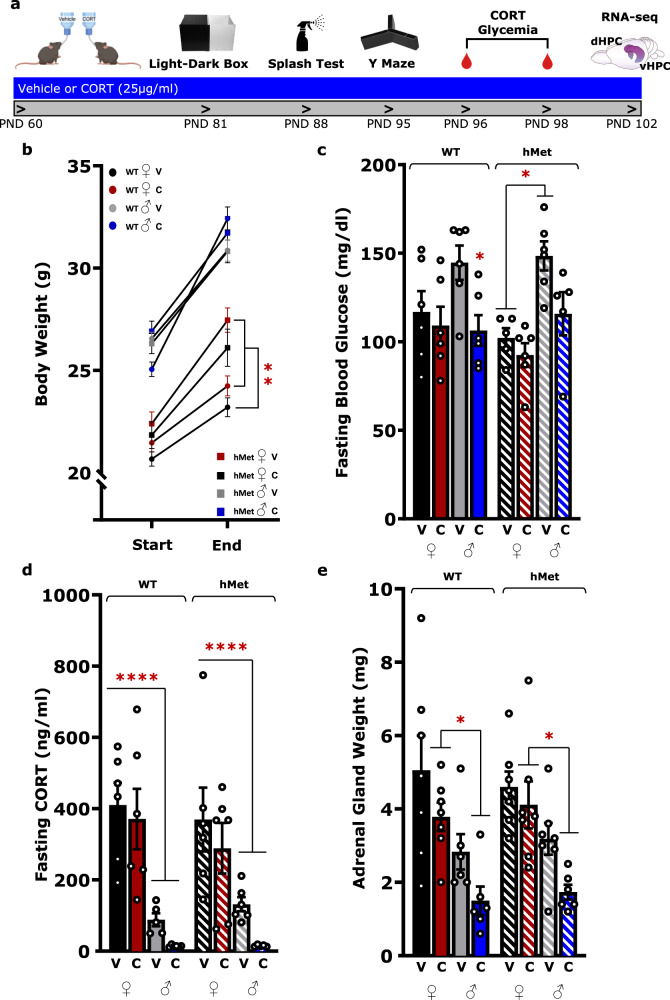
Endocrinological sex differences after chronic oral CORT. **a** Timeline for CORT treatment experiment. **b** Body weight recorded at the start and end of CORT treatment. Over the course of treatment, female hMet mice demonstrate a significant increase in body weight compared to WT under the same treatment (three-way ANOVA, treatment: *F*(1,85) = 7.457, *p* < 0.01, sex: *F*(1,85) = 9.093, *p* < 0.01, genotype: *F*(1,85) = 194.5, *p* < 0.0001, genotype × sex: *F*(1,85) = 14.96, *p* < 0.001). **c** Measures of blood glucose concentration after a 24-h fasting period. WT male mice showed a significant decrease in fasting blood glucose after CORT treatment while hMet male mice under vehicle had significantly increased fasting blood glucose levels compared to vehicle-treated hMet females (three-way ANOVA, treatment: *F*(1,38) = 10.81, *p* < 0.01, treatment: *F*(1,38) = 12.37, *p* < 0.01). **d** Measures of plasmatic CORT levels after a 24-h fasting period. Females showed an increased concentration of CORT compared to males across genotypes (three-way ANOVA, sex: *F*(1,38) = 51.85, *p* < 0.0001). **e** Adrenal gland weight at the day of sacrifice. After CORT treatment females had a significantly higher adrenal gland mass than males, regardless of genotype (three-way ANOVA, treatment: *F*(1,45) = 8.365, *p* < 0.01, sex: *F*(1,45) = 28.86, *p* < 0.0001). Columns represent the mean ± SEM of 5–12 determinations per group. *p < 0.05, **p < 0.01, ***p < 0.001, ****p < 0.0001. WT wildtype, hMet heterozygous for brain-derived neurotropic factor Val66Met, V vehicle, C/CORT corticosterone, ♀ female, ♂ male.

At 2 months of age, male and female WT or hMet mice were randomly assigned to either vehicle or CORT treatment. Drinking water was replaced with either a solution containing 25 µg/ml corticosterone (Sigma, St. Louis, MO) dissolved in 100% ethanol, and then diluted in regular tap water to a final concentration of 1%, or a solution of 1% ethanol in tap water (vehicle). Both corticosterone and vehicle solutions were replaced twice a week. Body weight was measured weekly on a standard scale over the course of CORT administration. Starting at 3 weeks of treatment, mice were tested in a behavioral battery that included the light–dark box, the splash test, and the Y maze test (Fig. 1a) (see Supplementary for detailed protocols). Behavioral assessment and tissue collection were performed in the second half of the light phase to exclude data interpretation biased by an acute effect of exogenous CORT. Plasmatic levels of CORT are similar in both CORT-treated mice and vehicle-treated mice during this phase [16].

### Blood glucose

Glucose levels were measured from blood samples after 5-weeks of CORT treatment during the dark phase, after a 24-h fasting period, and on the day of sacrifice (*n* = 5–6 mice/group). Collection occurred at 2:00 a.m. for dark phase testing, 8 days prior to sacrifice, using submandibular bleeding. Food was removed from the cage for 24 h prior to fasting blood collection, 4 days prior to sacrifice, using submandibular bleeding. For this method, we followed the IACUC guidelines for blood collection, namely, of the circulating blood volume, 10% of the total volume was safely removed every 2–4 weeks. On the day of sacrifice mice were rapidly decapitated and trunk blood was immediately collected. At both time points, one drop of blood was used to measure blood glucose with OneTouch UltraMini Blood Glucose Monitor (LifeScan, Zug, Switzerland). The remaining blood was collected for isolating plasma.

### Plasma CORT

Corticosterone levels were measured from plasma collected after 5 weeks of CORT treatment during the dark phase, after a 24-h fasting period, and on the day of sacrifice (*n* = 5–6 mice/group) using a Corticosterone Double Antibody RIA kit (MP Biomedicals Inc., Santa Ana, CA, USA). Briefly, blood was collected in K3 EDTA (K3E) 12 mg Blood Collection Tubes (BD Vacutainer, Franklin Lakes, NJ, USA). Samples were then centrifuged at 1000 × *g* for 10 min to collect plasma, which was rapidly frozen at −80 °C. Five microliter of plasma [diluted 1:200 in phosphosaline gelatin buffer (pH 7.0 ± 0.1)] and 100 μL of standard calibrators were incubated for 2 h with radioactive corticosterone I125 (7 μCi per vial) and then centrifuged at 1000 × *g* for 15 min. Radioactivity in the resulting precipitant was measured using a Hidex Automatic Gamma Counter (Turku, Finland). Corticosterone concentration was calculated using the count per minute (CPM) as a function of the logarithmic equation generated from the calibrators.

### Tissue collection and RNA-seq

After 6 weeks of treatment, mice were cervical dislocated, and brains were dissected for tissue collection. The dorsal (dHPC) and ventral hippocampi (vHPC) were isolated, immediately flash frozen, and stored at −80 °C. Four biological replicates were used per experimental group, comprising of RNA pooled from two animals each (see Supplementary for details). Quality control was performed on the reads obtained from the core and reads with a score of <15 were discarded [27, 28]. The reads were then aligned to the GRCm38 genome using the STAR aligner [29] with Ensemble annotation [30] and quantified to the gene-level using featureCounts [31]. These counts were analyzed using the R/Bioconductor framework [32] (www.R-project.org). Differentially expressed gene (DEG) analysis was conducted using the limma-voom package [33]. Overlaps between the differential expression of two independent RNA-seq comparisons were visualized and measured with “stratified” rank–rank hypergeometric overlap (RRHO) analysis [34] (see Supplementary for details). RNA-seq data have been deposited to GEO (GSE194059). DEGs are included in Supplementary Data 1. All other data are available upon request.

### Reverse transcriptase-quantitative polymerase chain reaction (RT-qPCR)

Total mRNA (*n* = 5–7 mice/group) was extracted from the vHPC using Qiagen Lipid Tissue Mini Kit (Qiagen, Germantown, MD, USA) and then reverse transcribed using mixed random primers and MuLV reverse transcriptase (Invitrogen). Quantitative real-time PCR was performed using SYBR Green master mix and a CFX Connect qPCR system (Bio-Rad) using primers specifically designed for Nr3c1 exons (Supplementary Table 1). *Gapdh* was used as a reference gene. Data are represented as 2^−ΔCt^.

### Methyl-DNA-immunoprecipitation assay

DNA from the vHPC (*n* = 5–7 mice/group) was extracted using Qiagen Lipid Tissue Mini Kit (Qiagen, Germantown, MD, USA) according to manufacturer’s instructions. DNA methylation levels were assessed by methyl-DNA-immunoprecipitation (meDIP) using the MagMeDIP kit (Diagenode, Denville, NJ) as previously described [35]. Primers were designed in the *Nr3c1* exons (Supplementary Table 1). MeDIP assay efficiency was assessed by RT-qPCR using internal positive and negative DNA controls (methylated/hydroxymethylated and unmethylated DNA) as well as control primers for testis specific H2B histone gene (which is methylated in all somatic cells but not in testis), and *Gapdh* promoter (which is poorly methylated), following manufacturer’s instructions. Data are represented as 2^−ΔCt^.

### Sequencing analysis and statistics

Behavioral, endocrine, mRNA levels, and DNA methylation (see Supplementary for details) data were analyzed using GraphPad Prism (GraphPad Software, Inc., USA) to perform a three-way ANOVA followed by Neumann–Keuls, post-hoc analysis. The *z*-score was calculated using the following formula: *z* = [(*x*−*x̅*_CONT_)/*σ*_CONT_]*(±1)], where *x* = behavioral variable and *σ* = standard deviation. Microsoft Excel (Microsoft, USA) was used to obtain gene expression profiles by sorting genes based on fold change.

## Results

### Endocrinological parameters in males and females after chronic oral CORT

Previous reports show that low dose oral CORT in males do not significantly change body weight but induce atrophy of the adrenal glands and increase levels of leptin, insulin, and plasmatic CORT at night [16]. We found that oral CORT did not affect body weight in either sex or genotype, however hMet females showed lower body weight than WT females regardless of treatment (*p* < 0.01) (Fig. 1b). Glycemia, measured after a 24-h fasting, was reduced in WT males under CORT (*p* < 0.05) but not in females, and no sex differences were observed at baseline. Yet, hMet females showed lower glycemia than hMet males with no effect of treatment (*p* < 0.05) (Fig. 1c). Levels of plasma CORT were also measured after 24-h fasting and showed that females displayed higher plasma CORT than males regardless of genotype, with no major changes after treatment (*p* < 0.001) (Fig. 1d). Glycemia and plasma CORT were also measured during the dark phase and at the end of the treatment, with no significant effect found at either timepoint (Supplementary Fig. 1a, b, e). Yet, during the dark phase, hMet male mice under CORT showed higher CORT than vehicle-treated hMet males (*p* < 0.001), an effect that was not observed in females of either genotype (Supplementary Fig. 1d). At the end of treatment, adrenal gland, uteri, and testes were dissected and weighed. Oral CORT increased the difference in adrenal gland weight between sexes, with females showing higher adrenal gland weight than males (*p* < 0.05) (Fig. 1e). However, no effect was observed after CORT on uterine and testes weight (Supplementary Fig. 1c, f). Therefore, while oral CORT did not affect body weight, it induced sex- and genotype-dependent effects on endocrinological parameters.

### Males and females exhibit opposite affective behavior after chronic oral CORT

**Fig. 2:**
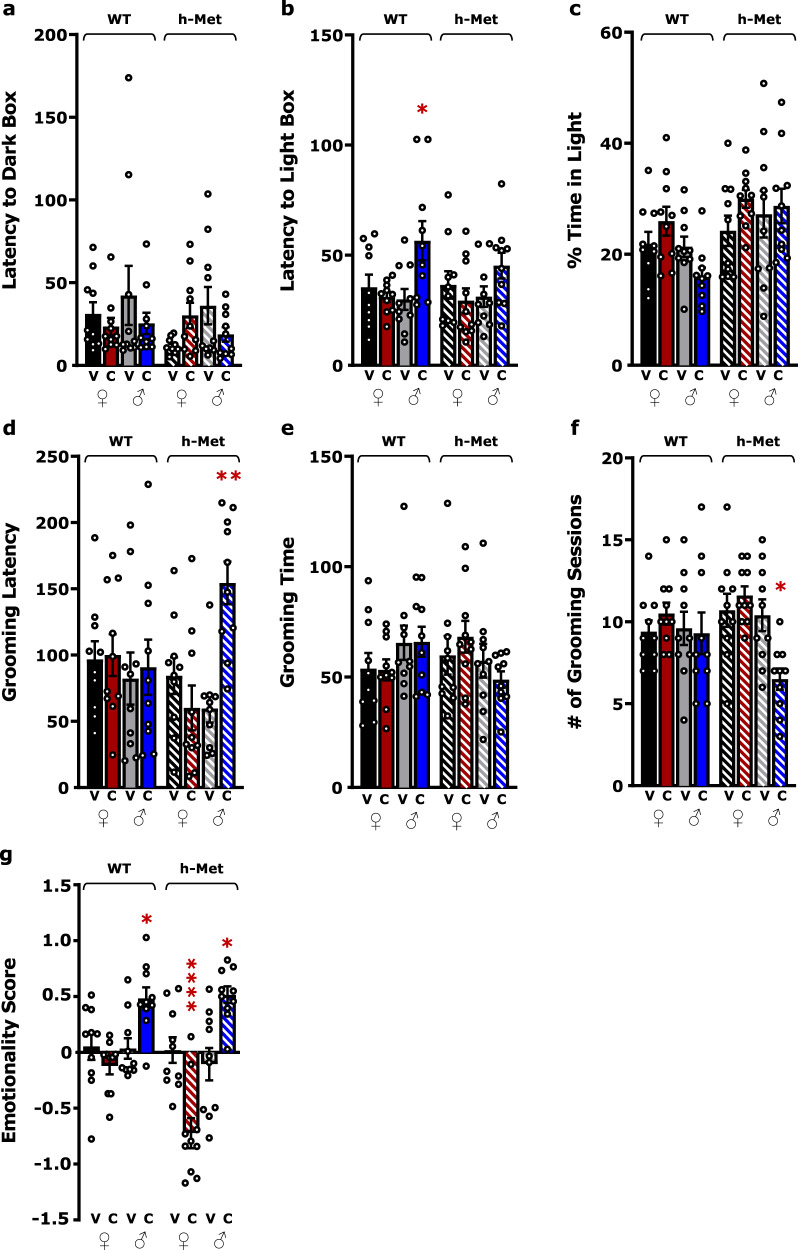
Measurements of affective behavior after CORT treatment show unique phenotypes in males and females. **a**–**c** Light–dark box test reveals increased anxiety-like behavior in male mice treated with CORT (three-way ANOVA, Latency to Light: treatment × genotype, *F*(1,72) = 10.31, *p* < 0.01; % Time in Light: sex *F*(1,72) = 10.31, *p* < 0.01). **d**–**f** Splash test reveals increased anxiety-like behavior in hMet mice treated with CORT (three-way ANOVA, grooming latency: genotype × sex: *F*(1,72) = 4.116, *p* < 0.05, treatment × genotype: *F*(1,72) = 7.351, *p* < 0.01, treatment × genotype × sex: *F*(1,72) = 6.163, *p* < 0.05; grooming time: genotype × sex: *F*(1,72) = 5.66, *p* < 0.05; # of grooming sessions: genotype: *F*(1,72) = 6.527, *p* < 0.05, treatment × genotype: *F*(1,72) = 6.125, *p* < 0.05). **g** Complementary variables of behavior across sex and genotype were compiled to calculate a *z*-score, which show that CORT-treated male mice displayed higher emotionality scores compared to vehicle-treated males, regardless of genotype, whereas CORT-treated hMet females displayed lower emotionality scores compared to vehicle-treated hMet females (three-way ANOVA, genotype: *F*(1,68) = 6.748, *p* < 0.05, sex: *F*(1,68) = 27.20, *p* < 0.0001, treatment × sex: *F*(1,68) = 36.72, *p* < 0.0001, treatment × sex: *F*(1,68) = 4.431, *p* < 0.05). Columns represent the mean ± SEM of 10 determinations per group. *p < 0.05, **p < 0.01, ***p < 0.001, ****p < 0.0001. PND post-natal day, WT wildtype, hMet heterozygous for brain-derived neurotropic factor Val66Met, V vehicle, C/CORT corticosterone, ♀ female, ♂ male.

To validate the behavioral phenotype in males and compare it with females, mice were assessed for anxiety- and depression-like behavior using the light-dark box test and the splash test. CORT increased the latency to enter the light box in WT males compared to vehicle-treated WT males (*p* < 0.05) but not in females of either genotype (Fig. 2b). No differences in latency to the dark box or percent time in light were found between groups (Fig. 2a, c). In the splash test, while no differences were found in grooming time across groups (Fig. 2e), only hMet males under CORT showed increased grooming latency (*p* < 0.01) and a decreased number of grooming sessions (*p* < 0.05) compared to vehicle-treated hMet males, and we found no differences in females of either genotype (Fig. 2d, f). However, when a *z*-normalization was applied across complementary variables of behavioral measurements [36], hMet females under CORT attained a negative behavioral score compared to hMet females under vehicle (*p* < 0.0001), indicating not only that CORT did not induce increased anxiety- or depression-like behavior in hMet females, but also that it had an opposite behavioral effect than in males (Fig. 2g). Thus, oral CORT was able to increase affective behavior in males (*p* < 0.05), but not in females, with hMet females displaying a behavioral phenotype that differed from all other groups. Cognitive performance was measured using the Y maze test. No differences in the discrimination ratio (preference for the novel arm), were found as function of sex, treatment, or genotype (Supplementary Fig. 2a–c).

### CORT induces a higher number of DEGs in the vHPC than the dHPC

**Fig. 3:**
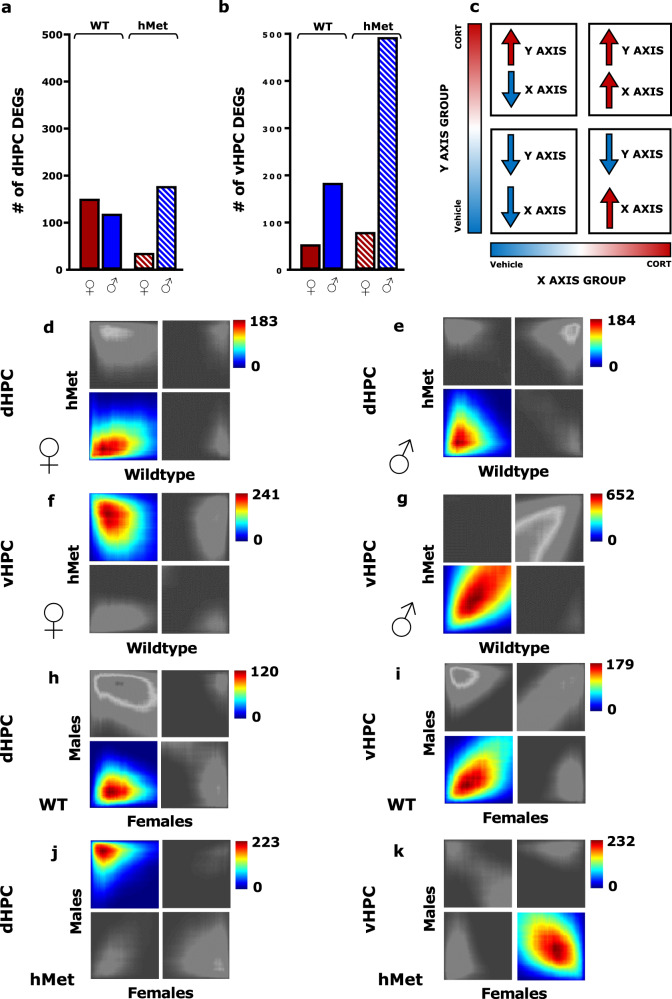
Genomic signatures in response to CORT along the dorsoventral axis of the HPC differ in males and females. The number of DEGs induced by CORT treatment within the **a** dorsal hippocampus and **b** ventral hippocampus. **c** Threshold free rank-rank hypergeometric overlap comparisons of DEGs. The lower left quadrant designates co-downregulated genes by CORT, upper right quadrant designates co-upregulated genes by CORT, and the upper left and lower right quadrants include oppositely regulated genes. Genes along each axis are listed from greatest to least significantly regulated from the outer to middle corners. Pixels represent the overlap between the transcriptome of each comparison as noted, with the significance of overlap [−log10(*p*-value) of a hypergeometric test] color coded. The quadrants insignificant to the analysis are shaded in gray. Comparison of WT and hMet DEGs in the dHPC in **d** females (max-log10(*p*-value) = 183) and **e** males (max-log10(*p*-value) = 184). Comparison of WT and hMet DEGs in the vHPC in **f** females (max-log10(*p*-value) = 241) and **g** males (max-log10(*p*-value) = 652). Comparison of WT female and WT male DEGs in the **h** dHPC (max-log10(*p*-value) = 120) and **i** vHPC (max-log10(*p*-value) = 179). Comparison of hMet female and hMet male DEGs in the **j** dHPC (max-log10(*p*-value) = 223) and **k** vHPC (max-log10(*p*-value) = 232). CORT corticosterone, WT wildtype, hMet heterozygous for brain-derived neurotropic factor Val66Met, ♀ female, ♂ male, DEG differentially expressed gene, dHPC dorsal hippocampus, vHPC ventral hippocampus.

We then investigated the genomic effects of CORT in the dHPC and vHPC, moving from recent evidence demonstrating that behavioral susceptibility in males correlates with genes implicated in stress coping [37]. DEGs (*p* < 0.05, FC > 1.3) in the dHPC and vHPC were identified using RNA-seq, and compared based on treatment, sex, and genotype (Supplementary Data 1). CORT induced similar levels of DEGs in the dHPC of WT males (119) and females (151), whereas hMet males exhibited a higher number of DEGs (178) than hMet females (36) (Fig. 3a). In the vHPC, CORT induced similar levels of DEGs in WT (54) and hMet females (80), albeit lower than both WT (184) and hMet males (493) (Fig. 3b). Notably, CORT induced a greater amount of DEGs in the vHPC (811) than in the dHPC (484).

Stratified RRHO test [34], a threshold-free approach that ranks genes by their *p*-value and effect size direction, was used to identify genes differentially regulated by CORT from a continuous gradient of significancy that were concordant across treatment, sex, and genotype in each brain region (Fig. 3c). Significant overlaps were identified using the point of highest −log10(*p*-value) from each quadrant as described in Plaiser et al. [38] (see Supplementary for details). Genes from WT and hMet mice converged in the dHPC in both sexes (Fig. 3d, e). In the vHPC, genes were discordant between WT females and hMet females (Fig. 3f). However, males showed concordance in the vHPC between genes in both WT and hMet mice (Fig. 3g). The patterns of overlap indicated that gene expression differences between WT and hMet mice were more prominent in the vHPC of female mice compared to males. An additional set of RRHOs was generated to compare the gene set of males and females in the dHPC and vHPC. Gene expression in WT mice converged across sexes in both the dHPC and vHPC (Fig. 3h, i). However, gene expression in hMet mice diverged across sexes in both the dHPC and vHPC (Fig. 3j, k). This indicates that the hMet variant drives sex differences in gene expression after CORT in both regions of the hippocampus.

Together, we found that CORT affects gene expression change predominantly in the vHPC, and that sex differences are more marked in the vHPC than in the dHPC, especially in hMet mice.

### CORT increases methylation of GR exons 1_C_ and 1_F_ in the vHPC of hMet females

**Fig. 4:**
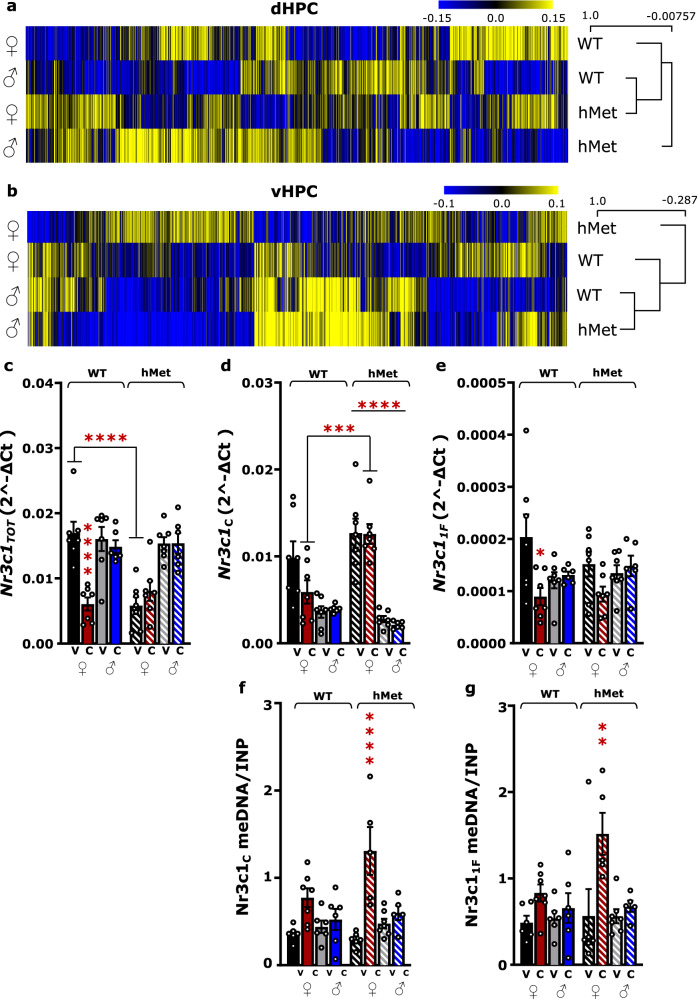
Epigenetic regulation of the glucocorticoid receptor in response to CORT. Heat map representing the normalized read densities of epigenetic and GR-binding genes across all groups in the **a** dHPC and the **b** vHPC. The cladogram in the right indicates the similarity in gene expression profiles, with average hierarchical linkage clustering based on Pearson correlation. Measurement of *Nr3c1* mRNA levels from the vHPC are expressed as 2^−ΔCt^ for **c** total *Nr3c1* mRNA as well as *Nr3c1* mRNA for **d** exon 1_C_ and **e** exon 1_F_. Total *Nr3c1* mRNA was significantly decreased in WT females after treatment with CORT. Baseline hMet females had lower total *Nr3c1* mRNA levels than matched WT females (three-way ANOVA, genotype: *F*(1,46) = 5.301, *p* < 0.05, treatment: *F*(1,46) = 5.928, *p* < 0.05, sex: *F*(1,46) = 37.66, *p* < 0.0001, sex × genotype: *F*(1,46) = 4.947, *p* < 0.05, treatment × genotype: *F*(1,46) = 12.71, *p* < 0.001, treatment × genotype × sex: *F*(1,46) = 8.730, *p* < 0.01). Levels of mRNA for *Nr3c1* exon 1_C_ in hMet females under CORT was significantly higher than hMet females at baseline, while hMet females had higher levels of exon 1_C_ mRNA than males regardless of treatment (three-way ANOVA, genotype: *F*(1,46) = 4.304, *p* < 0.05 sex: *F*(1,46) = 75.38, *p* < 0.0001, sex × genotype: *F*(1,46) = 13.64, *p* < 0.001). Levels of *Nr3c1* exon 1_F_ mRNA was significantly decreased in WT females after treatment with CORT (3-way ANOVA, treatment: *F*(1,46) = 5.384, *p* < 0.05, treatment × sex: *F*(1,46) = 9.458, *p* < 0.01). Measurement of *Nr3c1* **f** exon 1_C_ and **g** exon 1_F_ methylation levels from the vHPC are expressed as 2^−ΔCt^. CORT significantly increased levels of 5mC for *Nr3c1* exon 1_C_ and 1_F_ for hMet females (three-way ANOVA, exon 1_C_: three-way ANOVA, sex: *F*(1,40) = 4.875, *p* < 0.05, treatment: *F*(1,40) = 27.04, *p* < 0.0001, treatment × genotype: *F*(1,40) = 4.166, *p* < 0.05, treatment × sex: *F*(1,40) = 15.11, *p* < 0.001; exon 1_F_: sex: *F*(1,40) = 11.19, *p* < 0.05, treatment: *F*(1,40) = 11.19, *p* < 0.01, treatment × sex: *F*(1,40) = 5.394, *p* < 0.05). Columns represent the mean ± SEM of 5–7 determinations per group. *p < 0.05, **p < 0.01, ***p < 0.001. PND post-natal day, WT wildtype, hMet heterozygous for brain-derived neurotropic factor Val66Met, V vehicle, C/CORT corticosterone, dHPC dorsal hippocampus, vHPC ventral hippocampus.

Genomic sex differences in the dHPC and vHPC prompted the investigation on CORT-associated genes in males and females, based on evidence that CORT has genomic and epigenomic effects through the glucocorticoid receptor (GR) [2]. The analysis on genes that were concordant in the RRHOs was narrowed to investigate glucocorticoid receptor-binding genes (GRBGs) [39] and epigenetic modifiers [40]. Genes were clustered into separate heatmaps whose cladograms indicated concordance in CORT-induced gene expression across groups in the dHPC and vHPC (Fig. 4a, b). Both GRBGs and epigenetic modifiers clustered together in the vHPC of WT and hMet males, and they were weakly or not associated with WT females and hMet females, respectively. Thus, sex differences mainly drove the clustering of GRBGs and epigenetic modifiers. The same pattern of sex differences was not observed in the dHPC, where WT males and hMet females clustered instead, indicating that, in the dHPC, similarity in gene expression change after CORT was independent of sex and genotype. We then focused the investigation of sex differences in the vHPC by measuring the expression of the GR (encoded by *Nr3c1*) and its epigenetic regulation. Total *Nr3c1* mRNA was reduced in WT females under CORT (64%; *p* < 0.0001), as well as hMet females (*p* < 0.0001), but not in WT males or hMet mice of either genotype (Fig. 4c). Epigenetic regulation of *Nr3c1* was then assessed using RT-qPCR and meDIP on exons 1_A_, 1_C_, 1_D_, 1_F_, and 1_H_, whose methylation status is altered in several affective disorders in both animal models and clinical studies [41–46]. The mRNA levels of exon 1_C_ were increased in hMet females under CORT compared to CORT-treated WT females (*p* < 0.001), while levels in hMet females exceeded hMet males regardless of treatment (*p* < 0.0001) (Fig. 4d). The mRNA levels of exon 1_F_ were reduced in WT females under CORT (*p* < 0.05), but not in males (Fig. 4e). We then assessed the levels of 5-methylcytosine (5mC), a de novo enzymatic modification of the 5-position of the cytosine catalyzed by DNA methyltransferase proteins, which indicates epigenetic modification of DNA [47]. Levels of 5mC for exon 1_C_ were increased in hMet females under CORT (*p* < 0.0001), but not in males of either genotype (Fig. 4f). In addition, CORT increased 5mC levels of exon 1_F_ in hMet females (*p* < 0.01), but not in males of either genotype (Fig. 4g). No differences were found in levels of exons 1_A_, 1_D_, or 1_H_ as a function of sex, treatment, or genotype (Supplementary Fig. 3a–d). Therefore, we found that only females showed changes in methylation and mRNA levels of *Nr3c1* exons 1_C_ and 1_F_ in the vHPC in response to CORT, and that there is an epigenetic response to CORT in the vHPC that is unique to females carrying the BDNF Met variant.

## Discussion

We report, for the first time, behavioral sex differences in mice treated with non-invasive oral CORT, a paradigm that induces impaired activity of the HPA axis [16, 17]. Not only is this the first study investigating sex differences in anxiety- and depression-like behavior in the oral CORT model and in BDNF Val66Met mice, but the novelty also arises from the investigation of genomic signatures that depended on the hippocampal region analyzed, with the dHPC and vHPC expressing distinct pattern of gene networks, particularly GRBGs and epigenetic modifiers. We found that one epigenomic consequence of oral CORT was the methylation levels of the *Nr3c1* gene that differed in males and females as a function of the BDNF Met genotype.

As previously reported in males [16], we demonstrated that low-dose oral CORT did not induce changes in female body weight compared to controls, indicating that major metabolic consequences are indeed limited in both sexes. A trend in the atrophy of adrenal glands was only observed in males, consistent with studies showing that chronic treatment with CORT leads to smaller adrenal glands compared to controls [16], a phenomenon that is explained by adaptation of the adrenal gland at the cellular level [48], and that correlates with the blunted response to stress observed in this model [18]. Circulating plasma CORT levels measured after 24-h fasting were lower in males than in females, but treatment did not affect plasmatic CORT. This is consistent with evidence showing that mRNA and serum levels of corticosteroid-binding globulin are regulated by gonadal hormones, leading to sex differences in CORT levels [49–51]. High levels of glycemia reported in hMet males at baseline are consistent with previous findings showing insulin resistance in hMet mice [52]. Impairment in glucose regulation have also been observed in humans carrying BDNF Met variant that show insulin resistance and increased risk for metabolic disorders [53].

As reported in our findings, previous studies have also shown that oral CORT increases affective behavior in male mice of both genotypes [18, 54, 55]. Here we showed that WT females display no behavioral changes, consistent with previous findings on negative valence behaviors in WT females [56, 57], and that hMet females show instead an opposite effect on affective behavior compared to males. Other studies have investigated these behavioral phenotypes using exogenous CORT in males and found that prolonged CORT administration, independent of the administration route, produce both anxiety [58] and depression-like behavior [59]. In fact, a single acute injection of corticosterone was found to induce anxiety-like behavior 12 days later [60]. Females also display increased depression-like behavior in response to CORT injection, and effect that is not observed when CORT is delivered in drinking water [61].

We found that gene expression change after CORT was greater in the vHPC than in the dHPC in males of both genotypes and larger in the vHPC of males compared to females regardless of genotype. Curiously, in the dHPC, WT males and females treated with CORT showed comparable gene expression change, while hMet females under CORT displayed fewer DEGs compared to WT females. Accordingly, RRHOs showed genotypic differences in the vHPC of females, and discordant CORT-induced DEGs in both brain regions only in hMet males and females. This pattern of gene expression after CORT mirrored behavioral sex differences as well as the unique behavioral phenotype found in hMet females. Dorsoventral differences in gene expression have also been observed in other models of stress. For example, the immediate early gene *Fos* is primed for activation one month after traumatic stress in the vHPC but not the dHPC [62]. Six-week exposure to chronic mild stress increases the expression of FKBP5, a co-chaperone of GR, in the vHPC but not in the dHPC [6]. Others have shown that when chronic stress is combined with prenatal alcohol exposure, the expression of MR, GR, and type 1 CRH receptor is altered in a region-specific manner in the hippocampus and differs in males and females [63]. Interestingly, this evidence relating to GR gene expression compliments the sensitivity of the vHPC to glucocorticoid exposure [64, 65], and the importance of the GR in regulating neurotransmission in this brain region [66]. Collectively, previous data support our observations on the criticality of the vHPC in the response to exogenous CORT or other types of stressors.

It is worth noting that the distribution of GR in the hippocampus differs across subregions and subfields [5], a neuroanatomical difference that is also observed after exposure to exogenous CORT. Robertson et al. reported that GR expression is twice as high in the dHPC as in the vHPC in rats [67], a pattern that relates to differential GR expression in distinct hippocampal subfields in the presence of high levels of CORT [68]. Curiously, treatment with exogenous CORT in males has not been associated with changes in GR expression in the vHPC [69], in line with our measurements of mRNA levels in the vHPC of CORT-treated males.

These findings prompted us to investigate whether GR had different genomic effects in the dHPC and vHPC that intersected with sex and genotype. Notably, after translocating to the nucleus the GR interacts with number of epigenetic modifiers [70]. Thus, we also investigated whether changes in GR could be associated with differences in epigenetic regulation. Differential expression of GRBGs and several epigenetic modifiers (e.g., chromatin remodelers and histone acetyltransferases, demethylases, and methyltransferases) across groups showed that sex differences were observed in the vHPC but not in the dHPC where clustering was independent of sex and genotype. Interestingly, CORT-induced changes in the expression of GRBGs and epigenetic modifiers in hMet females differed substantially from the other groups, again reflecting the unique behavioral and genomic profile of hMet females. We have previously reported that the expression of GRBGs in CA3 pyramidal neurons of the hippocampus differs between WT and hMet male mice after stress [26]. Yet, DEGs from this hippocampal subregion in unstressed hMet mice largely overlap with gene expression changes observed in WT mice after stress [25]. Previous work from our group highlighted changes in GRBGs and a significant cluster of epigenetic modifiers in CA3 pyramidal neurons of adult offspring with a history of early life stress exposed to a second hit of stress in adulthood [71], suggesting an important role for the GR in the epigenetic signatures mediated by stress. While others also showed that exposure to exogenous CORT alter genome-wide epigenetic networks [72] as well as the expression of GRBGs [73] in the hippocampus, this is the first study demonstrating that these changes critically depend on the sex, genotype, and the hippocampal region that is investigated.

The relevance of exogenous CORT and its transcriptomic consequences is emphasized when reminded that up to 1/5 of the human genome is regulated by the GR [1]. Additionally, epigenetic modifications and DNA methylation of GR exons have been shown to reflect the stress status of an individual [45, 74]. These changes are often a consequence of adverse stimuli or overexposure to CORT. Variations in maternal licking and grooming in rats are associated with epigenetic modifications of the GR exon 1_7_ (corresponding to 1_F_ in humans and mice) that persist through adulthood [75]. In humans, methylation levels of *Nr3c1* are affected in offspring of prenatal depressive mothers [46] as well as subjects suffering from psychiatric disorders, including individuals with alcohol use disorder [35], victims of suicide [41, 44], combat veterans of PTSD [42], and women with bulimia nervosa [43]. Interestingly, these epigenetic modifications often occur on the exons 1_C_ and 1_F_ of the *NR3C1* gene. Here, we found that CORT increased DNA methylation of exons 1_C_ and 1_F_ of the *Nr3c1* gene selectively in the vHPC of hMet females under CORT, while the respective mRNA levels were unchanged (1_C_) or oppositely (1_F_) regulated in hMet females compared to WT females. In males, total mRNA levels and methylation of *Nr3c1*_*1C*_ *and Nr3c1*_*1F*_ were unaffected regardless of genotype. Interestingly, we found a negative correlation between levels of *Nr3c1*_*1C*_ methylation and emotionality score in hMet females under CORT (*r* = −0.966, *p* < 0.05) that did not exist in other groups (Table 1). This suggests that (i) a mechanism different than methylation controls the total expression of *Nr3c1* in CORT-treated WT females and males of either genotype; (ii) methylation in response to CORT at specific exons depends on genotype; (iii) the BDNF Met variant interacts with CORT to modulate methylation at discrete exons of *Nr3c1*.

**Table 1:** Pearson’s correlation coefficient associating the expression or methylation levels of *Nr3c1* to the behavioral *z*-score.

Group		*Nr3c1* mRNA			*Nr3c1* meDIP	
		Total	1_C_	1_F_	1_C_	1_F_
WT Vehicle ♀	Behavioral *z*-score	*p* = 0.469 *r* = 0.531	*p* = 0.854 *r* = 0.146	*p* = 0.234 *r* = −0.766	*p* = 0.106 *r* = 0.986	*p* = 0.173 r = 0.963
WT CORT ♀		*p* = 0.667 *r* = −0.083	*p* = 0.548 *r* = −0.363	*p* = 0.925 *r* = −0.059	*p* = 0.284 *r* = −0.601	*p* = 0.553 *r* = −0.359
hMet Vehicle ♀		*p* = 0.894 *r* = −0.368	***p*** = 0.015* ***r*** = **−0.946**	*p* = 0.303 *r* = −0.582	*p* = 0.154 *r* = 0.739	*p* = 0.699 *r* = 0.239
hMet CORT ♀		***p*** = 0.038* ***r*** = **−0.836**	*p* = 0.667 *r* = −0.226	*p* = 0.371 *r* = −0.450	***p*** = 0.034* ***r*** = **−0.966**	*p* = 0.133 *r* = −0.867

*WT* wildtype, *hMet* heterozygous for brain-derived neurotropic factor Val66Met, *V* vehicle, *C/CORT* corticosterone, ♀ female.

Pearson’s *r* and *p* values are detailed in the table. Significant correlations were found in females but not in males. *p < 0.05.

Bold values identify statistical significance (*p* < 0.05).

Glucocorticoids and BDNF share a complementary role in synaptic plasticity. Previous work showed that overexposure to CORT affects the signaling of BDNF in the hippocampus [76, 77], and that methylation changes of *Nr3c1* are linked to BDNF expression [78]. BDNF signaling not only modulates the excitability of the hippocampus but is directly impacted by fluctuations in the estrous cycle [79, 80]. In addition, CORT signaling is inhibited by estrogens, a mechanism that impairs the negative feedback on the HPA axis [2, 81]. Curiously, ovariectomy rescues impairment in object memory location in hMet females [25], which also show increased affective behavior compared to controls at discrete stages of the estrus cycle [82, 83] or in the presence of exogenous estradiol [84]. This suggests that the BDNF Met variant orchestrates complex gene pathways and behaviors that are unique to hMet females due to the distinctive intersection of CORT, BDNF, and estrogen signaling. Gomes de Assis and Gasanov (2019) recently discussed the implication of the BDNF and CORT integrative system in the BDNF Val66Met polymorphism [85], however further studies are needed to validate the mechanistic function of the BDNF Met variant and its distinct adaptive role in males and females.

Together, we showed that, in mouse models that recapitulate stress-related disorders, similar constructive validity (chronic oral CORT) is associated with opposed face validity (affective behavior) in males and females and suggests that distinct brain circuits and genomic regulation underlies behavioral outcomes in response to exogenous CORT. This opens a window for the investigation of biomarkers of stress that are both unique to genetically susceptible individuals and altered regardless of the behavioral phenotype that one may manifest.

## Supplementary information

Supplementary Methods

Supplementary Figures

Supplementary Data 1
