# Supplementary material for: Corticosterone induces discrete epigenetic signatures in the dorsal and ventral hippocampus that depend upon sex and genotype: focus on methylated Nr3c1 gene
Source: Transl Psychiatry. 2022 Mar 16;12:109. doi: 10.1038/s41398-022-01864-7 (PMC8927334; doi:10.1038/s41398-022-01864-7)
Supplement: Supplementary file 1 — Supplementary Methods [file 41398_2022_1864_MOESM1_ESM.pdf]

## SUPPLEMENTAL METHODS

### Light-Dark Box Test

The arena consisted of an open white-wall light box and a covered black-wall dark box (l=29cm, w=29cm). Mice started the test in the light box and were videotaped for 5 minutes by a camera fixed on the ceiling above the arena (50±10 lux). Time spent in the light box, latency to enter the dark box, and latency to reenter the light box were scored by an experimenter blind to the experimental groups and conditions. Mice that did not enter the dark box during the 5-minute test cut-off were excluded from the analysis and from further investigation.

### Splash Test

The splash test was performed as described by Santarelli et al.[1] and Isingrini et al.[2] with minor changes. After 30 minutes habituation to a new and empty testing cage, mice were then sprayed on the hindquarters with a 10% (w/v in water) sucrose solution, and immediately placed in the testing cage for 5 minutes (50±10 lux). Grooming behavior was videotaped by a camera fixed on the ceiling above the arena. The latency to the first grooming session, the total time grooming, and the number of grooming sessions were manually scored by an experimenter blind to the experimental groups and conditions. Mice that did not groom during the 5-minute test cut-off were excluded from the analysis and from further investigation.

### Y Maze Test

Mice were habituated for 45 minutes to an hour in testing room. Mice were tested using a 2-trial memory task in a Y-maze. The Y-maze consists of 3 identical arms illuminated by a dim light (30-70 lux) with mouse dimensions of l=40 cm, w=8.9 cm, and h=20 cm. The light intensity is the same in each arm and is higher in the center of the Y-maze. A camera fixed to the ceiling videotaped the arena. Visual cues, such as clothes with differing colors and patterns, were placed on the walls/curtains above each arm and kept constant throughout the behavioral testing. The floor of the maze was covered with bedding from the home cages of the mice that are being tested. In between each trial, the bedding was mixed and redistributed in order to eliminate olfactory cues. The task consisted of 2 trials separated by a 1-hour interval. During the 1st trial (acquisition phase), one arm of the Y-maze was closed, and animals were placed at the end of a familiar arm, facing the wall, and allowed to explore the two open arms for 10 minutes. During the 1-hour inter-trial interval mice were placed back into their home cages. During the second trial (test phase), mice had access to all three arms for 5 minutes. The time spent in the familiar arm, time spent in the novel arm, and number of center crossings were recorded and scored using Ethovision (Noldus Information Technology Inc., Leesburg, VA, United States). Arm discrimination index was calculated using the following formula: *discrimination index = (time in*

*novel arm – time in familiar arm)/(time novel arm + time familiar arm)*. The time spent in the novel arm (the one closed during the acquisition phase) was expressed as percentage compared to the percentage of random chance of exploration of the three arms (33% for each arm). Mice were included in the study only if the percentage of time spent in the novel arm was higher than 33%. Mice with >70% preference for one of the two arms in the acquisition phase were excluded from the analysis and from further investigation.

**Tissue Collection and RNA-Sequencing**

Mice were cervical dislocated and rapidly decapitated to extract whole brain. Brains were immediately dissected to isolate the dorsal (dHPC) and the ventral hippocampus (vHPC), and subsequently flash frozen and stored at -80 °C. Adrenal glands were dissected from the body, weighed, rapidly flash frozen and stored at -80 °C. RNA was extracted from the dHPC and vHPC tissue using Qiagen Lipid Tissue Mini Kit (Qiagen, Germantown, MD, USA). DNAse I treatment occurred during RNA extraction and RNA was examined by a Bioanalyzer (Agilent technologies, Santa Clara, USA). The left and right sides of the dHPC and vHPC were randomly included in a pool to minimize potential lateralization effects. In addition, the RNA from two different hippocampi from the same experimental group were also pooled[3-6] to achieve the necessary minimum yield for RNA-seq (>100ng/µl) and storage for further investigation of gene expression. The cDNA libraries were sequenced on an Illumina NextSeq 500 to obtain single-end 75-bp reads at an approximate sequencing depth of 35-40 million reads per sample.

**Rank-Rank Hypergeometric Overlap**

The full threshold-free lists of differential expression data were first ranked by increasing log fold change. The RRHO2 "stratified" method (https://rdrr.io/github/RRHO2/RRHO2/) was used to detect the overlap between genes differentially expressed in the same or opposite directions[7], where the bottom left and top right quadrant display overlaps of genes with concordant differential expression, and the top left and bottom right display discordant overlap. Threshold-free differential expression lists were ranked using the -log10(p-value) corresponding to the sign of the full change value generated from the limma-voom package. The point from each quadrant that had the highest absolute log10-transformed significance denoted the rank thresholds (and accordingly the metric thresholds) on the x- and y-axis (i.e. in both models) that yields the most statistically significant set of overlapping differentially expressed genes or co-regulated genes[8].

**Sample Size Determination**

Sample size was established from power calculations of expected effect sizes for the experimental cohorts and from previous experience with the following equation:

$$N = [2*(\text{standard deviation})^2 * (\text{power index})] / (\text{change in mean})^2$$

*power index equals 10.5 for alpha (significance level) = 0.05 and for beta (chance of type II error) = 0.1*

## REFERENCES


1. Santarelli L, Saxe M, Gross C, Surget A, Battaglia F, Dulawa S, et al. Requirement of hippocampal neurogenesis for the behavioral effects of antidepressants. Science. 2003;301(5634):805-9.
2. Isingrini E, Camus V, Le Guisquet AM, Pingaud M, Devers S, Belzung C. Association between repeated unpredictable chronic mild stress (UCMS) procedures with a high fat diet: a model of fluoxetine resistance in mice. PLoS One. 2010;5(4):e10404.
3. Anand S, Mangano E, Barizzone N, Bordoni R, Sorosina M, Clarelli F, et al. Next Generation Sequencing of Pooled Samples: Guideline for Variants' Filtering. Scientific Reports. 2016;6(1):33735.
4. Bansal V, Tewhey R, Leproust EM, Schork NJ. Efficient and cost effective population resequencing by pooling and in-solution hybridization. PloS one. 2011;6(3):e18353-e.
5. Rellstab C, Zoller S, Tedder A, Gugerli F, Fischer MC. Validation of SNP allele frequencies determined by pooled next-generation sequencing in natural populations of a non-model plant species. PloS one. 2013;8(11):e80422-e.
6. Rivas MA, Beaudoin M, Gardet A, Stevens C, Sharma Y, Zhang CK, et al. Deep resequencing of GWAS loci identifies independent rare variants associated with inflammatory bowel disease. Nat Genet. 2011;43(11):1066-73.
7. Cahill KM, Huo Z, Tseng GC, Logan RW, Seney ML. Improved identification of concordant and discordant gene expression signatures using an updated rank-rank hypergeometric overlap approach. Sci Rep. 2018;8(1):9588.
8. Plaisier SB, Taschereau R, Wong JA, Graeber TG. Rank-rank hypergeometric overlap: identification of statistically significant overlap between gene-expression signatures. Nucleic Acids Res. 2010;38(17):e169.
