# Supplementary material for: Corticosterone induces discrete epigenetic signatures in the dorsal and ventral hippocampus that depend upon sex and genotype: focus on methylated Nr3c1 gene
Source: Transl Psychiatry. 2022 Mar 16;12:109. doi: 10.1038/s41398-022-01864-7 (PMC8927334; doi:10.1038/s41398-022-01864-7)
Supplement: Supplementary file 2 — Supplementary Figures [file 41398_2022_1864_MOESM2_ESM.pdf]

Primer Sequences.

Gene Symbol	Forward	Reverse
Gapdh	5′-AACAGCAACTCCCACTCTTC	5′-CCTGTTGCTGTAGCCGTATT
Nr3c1 mRNA		
**Total**	5′- CAAGGGTCTGGAGAGGACAAC	5′-TGCTGTGGAGGAGCTGGA
**1_A**	5′-ACTCTGCGTAAGAATGGAGAAG	5′-GAAACATCTTCCTGGCTGAGA
**1_C**	5′-GCGACTGTTGACTTCCTTCT	5′-CCACCGCAGCCAGATAAA
**1_D**	5′-CTCCGATCAGAAGTGCCAAG	5′-GGGAGTAAGGTGTTATGGTGTT
**1_F**	5′-CCAGGGAGAAGAGAAACTAAAGAAACT	5′-CGCGGCTTCTTGGCCTTT
**1_H**	5′-TCAGGAATTGCGGCCTTAC	5′-TGCTCCCTTAAGCGACATTTAT
Nr3c1 Methylated DNA Immunoprecipitation		
**1_A -32580 - 32478 bp**	5′-GGGTGGGTCAGAAGACTAAGA	5′-AGGGATCAGCAGAGCAGATAA
**1_C -2424 -2333 bp**	5′-CTTGTCAGCCGGGAACG	5′- CACGGAGAAGGAAGTCAACAG
**1_F -3267 -3186 bp**	5′-GTGTCTGCCGTCCTGTG	5′-AGTTGCGCGAAGTGTGT

Supplementary Table 1

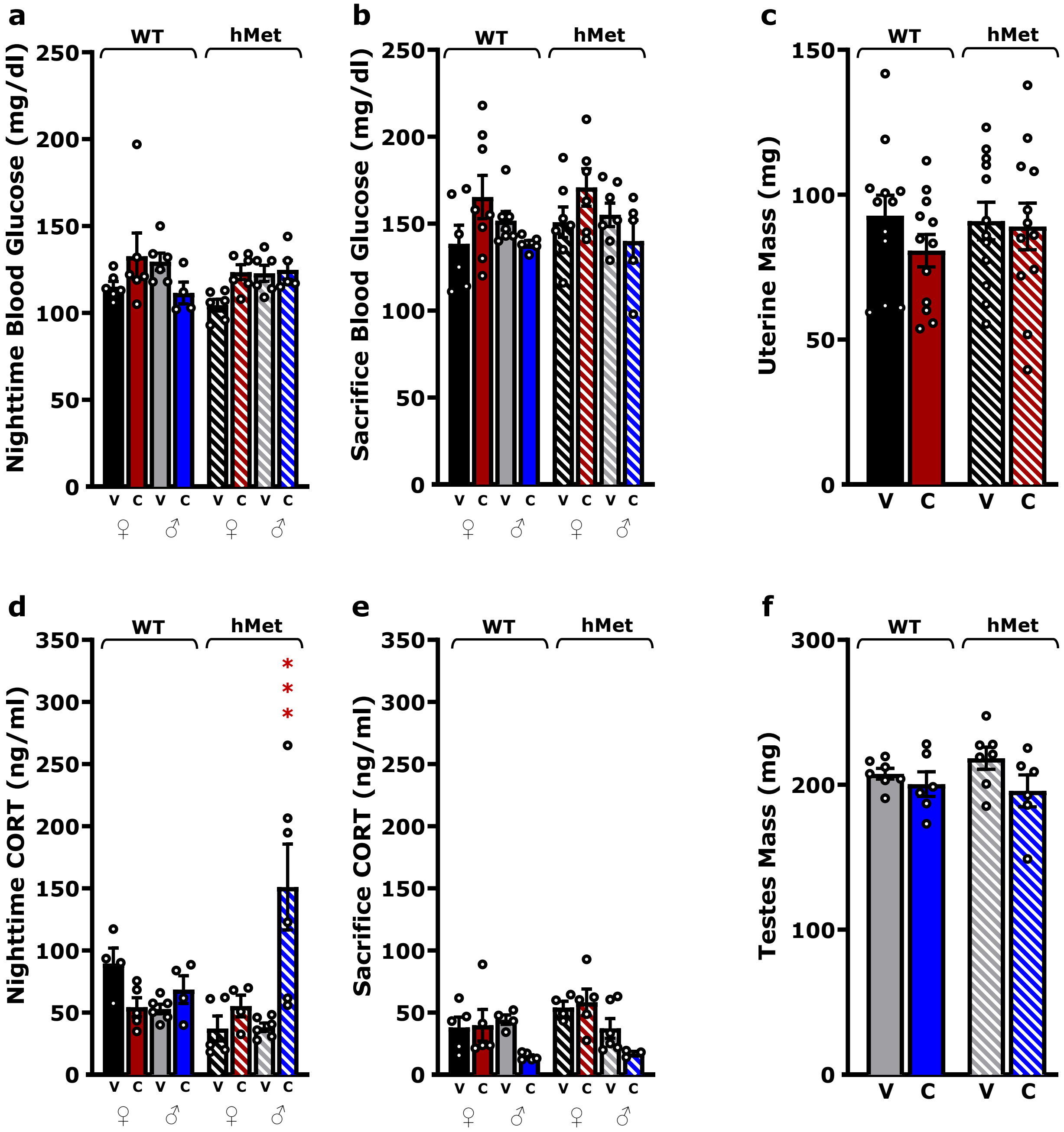

Supplementary Fig. 1

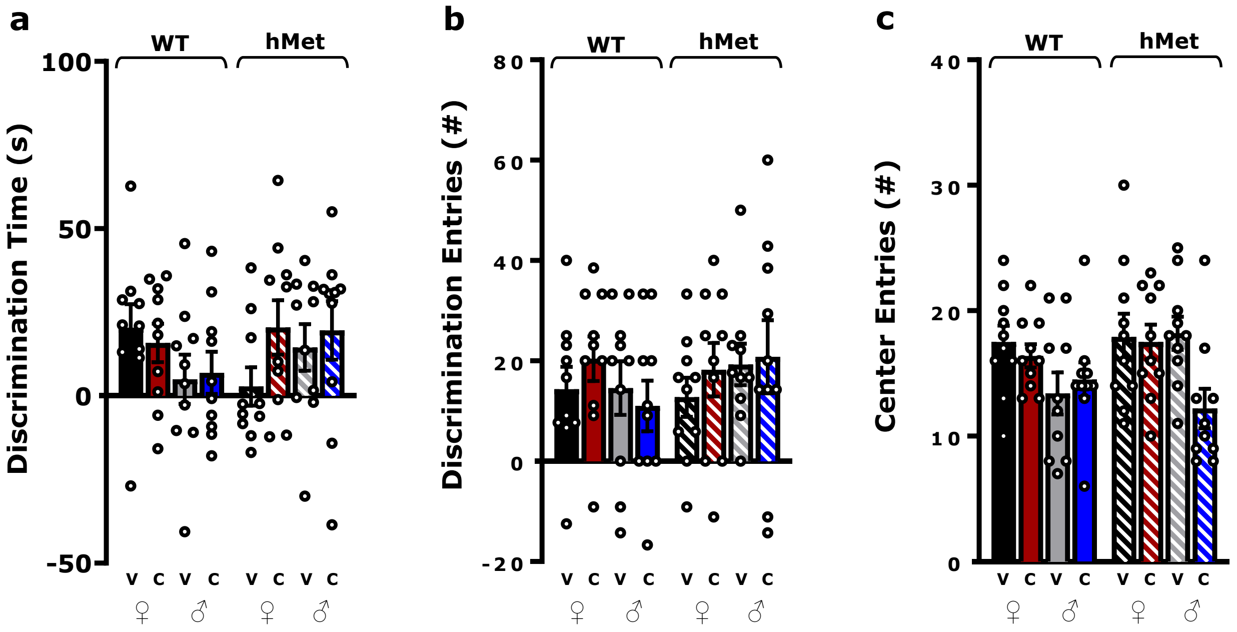

Supplementary Fig. 2

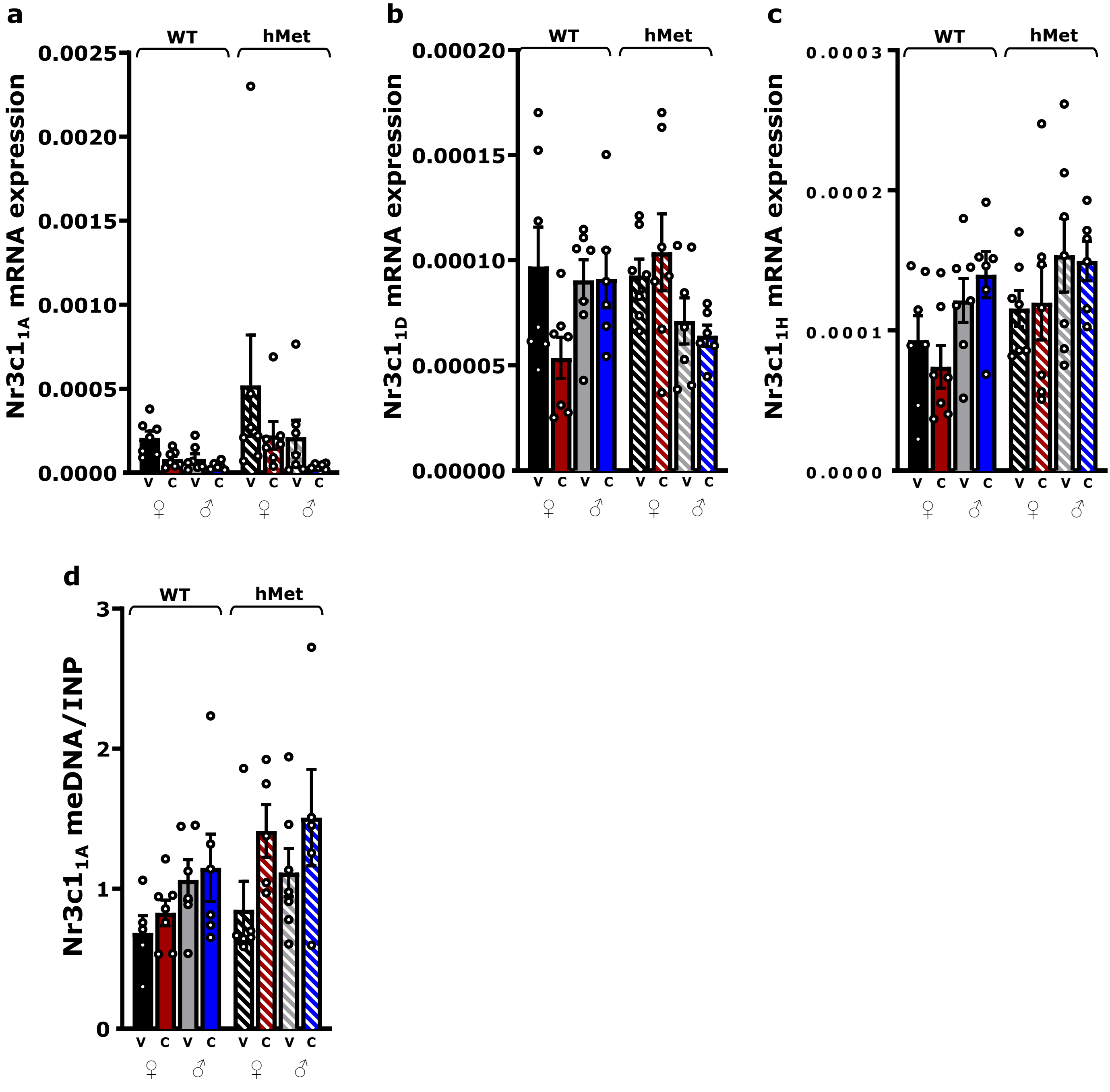

Supplementary Fig. 3

SUPPLEMENTARY LEGENDS

Supplementary Figure 1. Metabolic Signatures at Nighttime and Sacrifice Measures of blood glucose concentration were obtained a) during the night phase and b) at the point of sacrifice (3-way ANOVA, nighttime glucose: treatment x sex: $F(1,37) = 7.871$, $p<0.01$; sacrifice glucose: treatment x sex: $F(1,43) = 7.677$, $p<0.01$). Measures of blood CORT concentration were obtained d) during the night phase and e) at the point of sacrifice. During the night phase, male hMet mice had significantly increased CORT levels after treatment (3-way ANOVA, nighttime CORT: treatment: $F(1,32) = 5.470$, $p<0.05$, sex x genotype: $F(1,32) = 6.290$, $p<0.05$, treatment x sex: $F(1,32) = 9.333$, $p<0.01$, treatment x genotype: $F(1,32) = 10.01$, $p<0.05$; sacrifice CORT: sex: $F(1,29) = 10.24$, $p<0.01$, treatment x sex: $F(1,29) = 5.369$, $p<0.05$). Measurement of c) uterine mass and f) testes mass at time of sacrifice. Columns represent the mean ± S.E.M. of 3-12 determinations per group. *$p < 0.05$, **$p < 0.01$, ***$p < 0.001$, ****$p < 0.0001$. WT: wildtype, hMet: heterozygous for brain-derived neurotropic factor Val66Met, V: vehicle, C/CORT: corticosterone, ♀: female, ♂: male.

Supplementary Figure 2. CORT has no effect on spatial memory task a-c) The Y-maze test reveals that CORT treatment had no effect on spatial memory performance (3-way ANOVA, center entries: sex: $F(1,72) = 7.153$, $p<0.01$). Columns represent the mean ± S.E.M. of 10 determinations per group. WT: wildtype, hMet: heterozygous for brain-derived neurotropic factor Val66Met, V: vehicle, C/CORT: corticosterone, ♀: female, ♂: male.

Supplementary Figure 3. Expression of mRNA and methylation of *NR3C1* Measurement of vHPC mRNA levels as 2^-ΔCt for *Nr3c1* a) exon 1_A, b) exon 1_D, and c) exon 1_H (3-way ANOVA, exon 1_D mRNA: sex x genotype: $F(1,46) = 6.458$, $p<0.05$; exon 1_H mRNA: genotype: $F(1,46) = 4.190$, $p<0.05$, sex: $F(1,46) = 9.009$, $p<0.01$). d) Measurement of NR3C1 exon 1_A methylation levels from the vHPC are expressed as 2^-ΔCt (3-way ANOVA, genotype: $F(1,39) = 4.421$, $p<0.05$, treatment: $F(1,39) = 4.599$). Columns represent the mean ± S.E.M. of 4-7 determinations per group. *$p < 0.05$, **$p < 0.01$, ***$p < 0.001$. PND: post-natal day, WT: wildtype, hMet: heterozygous for brain-derived neurotropic factor Val66Met, V: vehicle, C: corticosterone, ♀: female, ♂: male.
